# Highly-Selective Analytical Strategy for 90 Pesticides and Metabolites Residues in Fish and Shrimp Samples

**DOI:** 10.3390/molecules28104235

**Published:** 2023-05-22

**Authors:** Yage Guo, Jun Xie, Fengshou Dong, Xiaohu Wu, Xinglu Pan, Xingang Liu, Yongquan Zheng, Jie Zhang, Jun Xu

**Affiliations:** 1 State Key Laboratory for Biology of Plant Diseases and Insect Pests, Institute of Plant Protection, Chinese Academy of Agricultural Sciences, Beijing 100193, China; 2 Institute of Zoology, Chinese Academy of Sciences, Beijing 100101, China

**Keywords:** aquatic products, pesticide, residue, SPE, HPLC-MS/MS

## Abstract

The analysis of pesticide residues in aquatic products is challenging due to low residue levels and the complex matrix interference. In this study, we developed a simple, fast method for the trace analysis of 90 pesticides and metabolites in aquatic products. The analytes covered a wide polarity range with log Kow (log octanol-water partition coefficient) ranging from −1.2 to 6.37. Grass carp (*Ctenopharyngodon idellus*) and prawn (*Penaeus chinensis*) samples were chosen to validate the quantification method. The samples were extracted by 0.2% formic-acetonitrile, cleaned by solid-phase extraction (PRiME HLB), and analyzed by high performance liquid chromatography−tandem mass spectrometry. The results showed good linearities for the analytes and were observed in the range of 0.05–50 μg/L. The recoveries of the method were within 50.4–118.6%, with the relative standard deviations being lower than 20%. The limits of quantifications (LOQs) of the method were in the range of 0.05–5.0 μg/kg, which were superior to values compared with other research. The developed method was applied to detect pesticide residues in prawn samples from eastern coastal areas of China. Three herbicide residues of diuron, prometryn, and atrazine were detected in prawn samples. The method was sensitive and efficient, which is of significance in expanding the screening scope and improving the quantitative analysis efficiency in aquatic products.

## 1. Introduction 

Pesticides play a key role in enhancing crop production and controlling pests. However, the extensive use of pesticides in modern agriculture has resulted in agricultural nonpoint source pollution causing risk hazards for water [1,2]. Pesticides can enter the aquatic environment through various ways, such as drift, irrigation, wash-off, drainage, and container and equipment cleaning practices [3]. These pesticides in the aquatic ecosystems may cause adverse effects on water pollution and the diversity of aquatic biota. More importantly, these pesticides in water could bioaccumulate in non-target species and even amplify at higher concentrations along the food chain; ultimately, they could cause risks to humans [1,4].

In aquatic products, fish and shrimp, the main providers of high-quality protein, polyunsaturated fatty acids, and vitamins and minerals, are an important part of a healthy diet [5,6]. Nonetheless, they can also be exposed to pesticides. For example, fish species are at a higher nutritional level in the aquatic food chain and easily bioaccumulate pesticides [3], and shrimp are considered benthic organisms that easily accumulate pesticides from sediments [7,8,9]. Therefore, it is very vital to develop analytical methods to detect pesticide residues in aquatic products for health reasons or ecological reasons [10].

In the past, and presently, the top concerns were relevant to the analytical methods of persistent organic pollutants because of their specific semi-volatile, persistent, and bio-accumulative characteristics [11,12,13,14,15,16,17,18]. As for the currently used pesticides, a comparison of the method with other methods in terms of analyte species, analysis time, sample pretreatment method, and LOQs is shown in Table 1. Previous research has focused on a limited number of pesticides [19,20,21,22] or has been restricted to a certain class of pesticides, such as pyrethroids pesticides [23], organofluorine pesticides [24], and triazine herbicides [25], which is a disadvantage for the large-scale screening of pesticide residues in aquatic products. In this study, 90 pesticides and metabolites were selected, including 45 insecticides, 18 fungicides, 23 herbicides, 2 plant growth regulators, and 2 acaricides. More importantly, metabolites with toxicity were chosen and analyzed. For example, phorate (the definition of the residue for compliance with the MRL and for dietary risk assessment for plant and animal commodity) content is defined as the sum of phorate and its oxygen analogue, and their sulfoxides and sulfones, expressed as phorate [19]. Likewise, the total residual amounts of fipronil include the concentration of fipronil, fipronil-sufone, fipronil-sufoxide, and fipronil-sufoxide [19]. Furthermore, fipronil-sulfone was found to be 3.3 times more toxic to bluegill sunfish and 6.6 times more toxic to freshwater invertebrates than fipronil [20]. The degradation products of atrazine are mainly desethylatrazine and hydroxyatrazine, which are 2–10 times more hazardous than the parent atrazine [21]. 

In the meantime, most of the analysis time was longer than 20 min. In the study, analysis time was reduced to 17 min for 90 pesticides and metabolites, which greatly improved work efficiency. Aquatic products are relatively complex matrices with a high protein and lipid content. The protein and fat can be extracted by organic solvent and this produces a strong matrix effect (ME) affecting the test results [4]. The methods of fat removal mainly include liquid–liquid extraction (LLE), gel permeation chromatography (GPC), solid-phase extraction (SPE), and dispersive solid-phase extraction (d-SPE) [22]. Among these methods, Maia et al. employed LLE for purification of the samples [8], while Chang et al. employed GPC for the purification of the samples [9]. Although these methods were effective in removing fat, they were time-consuming and inefficient. Most of the reported methods in the purification of aquatic products have focused on the d-SPE method with primary secondary amine (PSA) and octadecylsilane (C18) adsorbents. Although it had some advantages in terms of improving efficiency, it was still not effective in eliminating fat. Therefore, the LOQs of the methods were mainly above 1.0 μg/kg. Some studies have also optimized new absorbents (Z-Sep, mZrO_2_@Fe_3_O and so on) instead of PSA and C18. However, these adsorbents were not available. This study employed PRiME HLB for the purification of the samples, which had significant advantages in removing fat and showed lower LOQs in comparison to previous methods. The LOQs of the method were as low as 0.05 μg/kg, which can be used to monitor pesticide residues in aquatic products at trace levels.

In this study, an analysis method concerning multi-residue in fish and shrimp was developed. These pesticides were developed for 77 pesticides and 13 metabolites. The different extraction and purification methods were optimized to obtain precise and accurate results. The developed method was validated according to the SANTE guidelines by the European Union, in terms of linearity, recovery, relative standard deviation (RSD), LOQ, and ME [23]. The optimized method was successfully applied to analyze prawn samples, which provided a basis for detecting multi–pesticide residues in aquatic products at trace levels. 

**Table 1:** Comparison of this method to other sample methods for analysis of pesticides and metabolites in aquatic products.

Category	Compound	Aquatic Biota	Analysis Time	Extraction	Cleanup	Instrument	LOQ (µg/kg)	References
42 7	pesticides PCBs	fish	38	ACN	MSPE: mZrO_2_@Fe_3_O_4_)	GC-MS/MS	0.02–4.40 *	[14]
18 37 13	pesticides PCB/PAH/PDBE flame retardants	catfish	9.025	ACN	dSPE: Z-Sep	GC-MS/MS	0.5–5.0	[16]
15	pyrethroids	tilapia, tainha	25	ACN	dSPE:C18, PSA and Z-Sep	GC-MS	5.0 or 10.0	[24]
6	organofluorine	grass carp, white shrimp, European eel	14.5	ACN	SPE: NH_2_	LC-MS/MS	0.3–0.5	[25]
14	triazine	bivalves	20.5	4% acidic ACN	d-SPE:MIPs	GC-MS/MS	0.1–1.59	[26]
32	pesticides	flatfish, eel, oyster, and shrimp	27	1% FA/ACN	d-SPE: PSA, C18	LC-HRMS	0.5–5.0	[27]
52	pesticides	fish	25	ACN	d-SPE: PSA, C18	LC-MS/MS	0.04–49.8	[28]
13	pesticides	roach, perch and carp	17	ACN	Hexane LLE	LC-MS/MS	1.0–7.4	[29]
33	pesticides	*Cyprinus carpio*	20	Ethylacetate/ACN(1:1)	GPC	LC-MS/MS	0.003–0.247	[30]
22	pesticides and metabolites	freshwater fish marine fish	8	1%acetic acid/ACN.	SPE+dSPE	LC-MS/MS	0.5–5.0	[22]
20	pesticides	Eel and shrimp	30	ACN	d-SPE: PSA, C18	LC-MS/MS	<5.0	[31]
67	pesticides	fish	33	1% acidic ACN	d-SPE: PSA, C18	LC-MC/MC	1.0–15.0	[32]
90	pesticides and metabolites	fish, shrimp	17	0.2% FA/ACN	SPE: PRiME HLB	LC-MS/MS	0.05–5.0	this method

* refer to LOD.

## 2. Results and Discussion

### 2.1. Optimization of the LC–MS/MS

**Figure 1:**
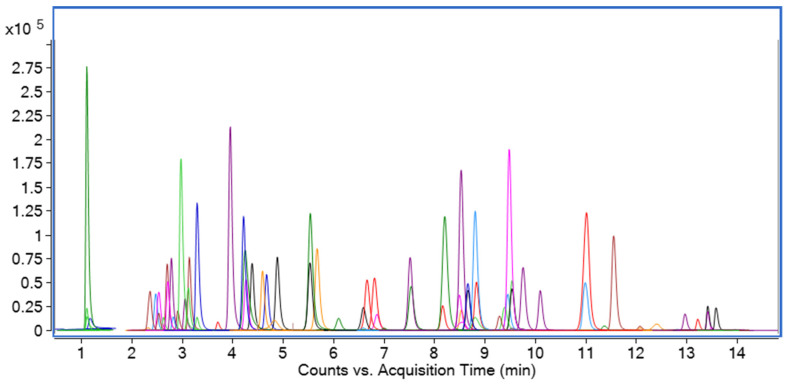
MRM chromatograms of 90 pesticides and metabolites obtained by HPLC–MS/MS.

For the analysis of 90 pesticides and metabolites, a standard solution of each analyte (10 μg/L) was prepared and directly injected into the autosampler to obtain the optimal mass spectrum conditions. The MS/MS conditions of each analyte were optimized through the following pattern: MS2 SCAN mode, SIM mode, Product ion mode, and MRM mode. Among the analytes, 83 analytes were detected using the ESI positive ion mode and 7 analytes were detected using the ESI negative ion mode. The multiple reaction monitoring (MRM) transitions, retention time, fragmentor, and collision energy of each analyte are summarized in Table S1. For the mobile phase, water was chosen as the aqueous phase because of the presence of the ESI negative ion mode. The MRM chromatograms of 90 pesticides and metabolites were shown in Figure 1. 

The dynamic multiple reaction monitoring (dMRM) data acquisition mode can yield a higher response, symmetrical peak shape because the mass spectrometer scans only certain channels within a specified time window so that more scans can be obtained [33,34]. In this study, the MRM mode and dMRM mode were compared. As shown in Figure S1, the dMRM mode indicated a 3- to 10-fold higher response than the MRM mode. Therefore, the dMRM mode was used to achieve a high sensitivity and superior peak shape.

### 2.2. Optimization of Sample Preprocessing

#### 2.2.1. Extracting Procedure

**Figure 2:**
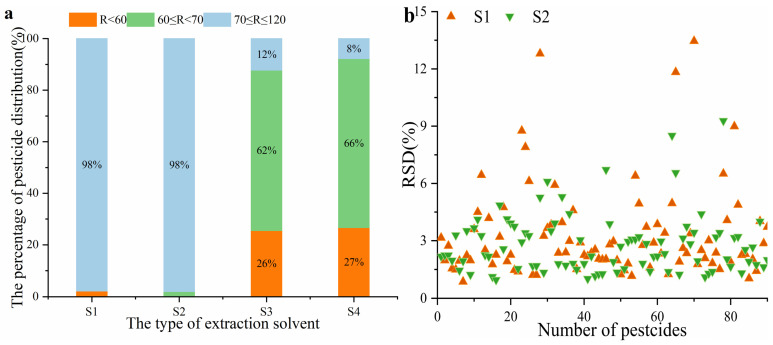
Effect of sample extraction: (**a**) The recoveries distribution of 90 pesticides and metabolites under different types of extraction solvents (S1: ACN; S2: 0.2% FA/ACN; S3: ACN/water (8:2); S4: ACN/water (8:2) with 0.2% FA). (**b**) The RSDs of 90 pesticides and metabolites under different types of extraction solvents ((S1: ACN; S2: 0.2% FA/ACN).

The choice of extraction solvents was significant in obtaining a satisfactory recovery. In the residue analysis, acetonitrile (ACN), ethyl acetate, and acetone were the common extraction solvents. However, ACN was chosen as the preferred solvent because it extracted fewer coextracted matrix components [22]. Meanwhile, it was also the major component of the mobile phase and could be directly injected into the column without evaporation and reconstitution [35]. In this study, the extraction efficiencies were examined using pure ACN, 0.2% formic acid/ACN (FA/ACN), ACN/water (8:2), and ACN/water (8:2) with 0.2% FA. ACN/water (8:2) was selected because it had been used to analyze pesticide residues in fishery products [10]. The results of different extraction solvents are shown in Figure 2a. The recoveries of 90 pesticides and metabolites using ACN/water (8:2) and ACN/water (8:2) with 0.2% FA were not satisfactory, and the recoveries of almost 26% and 27% of analytes were less than 60%. The recoveries of two analytes (methamidophos and hydroxyatrazine) using ACN were less than 60%. The addition of formic acid in ACN improved the recovery of methamidophos (74%) and hydroxyatrazine (64%). The RSDs of ACN and 0.2% FA/ACN as extraction solvents are compared in Figure 2b. Furthermore, smaller RSDs were observed when 0.2% FA/ACN was used as the extracting solvent. Thus, 0.2% FA/ACN was selected as the optimal extraction solvent for the target analytes.

#### 2.2.2. Clean-Up Conditions

**Figure 3:**
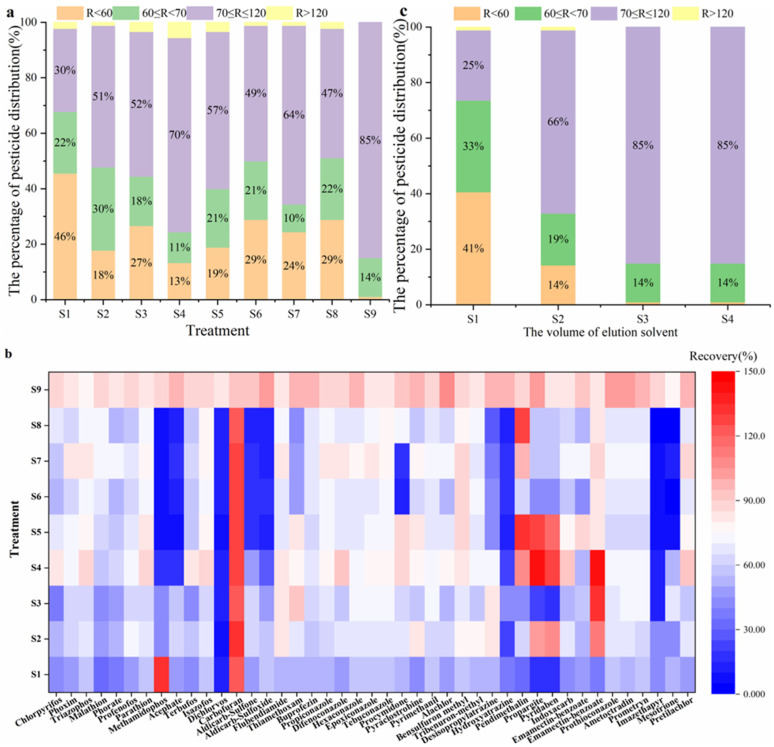
Effect of sample cleanup: (**a**) Recoveries distribution of 90 pesticides and metabolites under different types of cleanup conditions (S1–S4: Cleanert PEP (sorbent), S1: MEA: Water = 8:2 (loading solvent), MEA (elution solvent); S2: MEA: Water = 8:2 (loading solvent), ACN (elution solvent); S3: ACN: Water = 8:2 (loading solvent), MEA (elution solvent); S4: ACN: Water = 8:2 (loading solvent), ACN (elution solvent); S5–S8: Oasis HLB, S5: MEA: Water = 8:2 (loading solvent), MEA (elution solvent); S6: MEA: Water = 8:2 (loading solvent), ACN (elution solvent); S7: ACN: Water = 8:2 (loading solvent), MEA (elution solvent); S8: ACN: Water = 8:2 (loading solvent), ACN (elution solvent); S9: PRiME HLB (sorbent), ACN (elution solvent)); (**b**) recoveries of target compounds under different types of cleanup conditions; (**c**) recoveries of distribution of 90 pesticides and metabolites with different elution volumes.

Aquatic samples were rich in fat components, and they had an adverse impact on quantitative results. In this study, different types of SPE were tested for recovery. In the initial experiment, SPE cartridges (the Oasis HLB cartridge and Cleanert PEP cartridge), which belonged to hydrophilic–lipophilic balanced copolymer, were selected for the purification of the 90 analytes [4]. It was previously reported that the efficiency of SPE was determined by the variety of sorbents, type of the loading solvents and elution solvents, etc. [29,36]. Therefore, different varieties of combinations (including the variety of sorbents, type of the loading solvents and the elution solvents) were designed to optimize several relevant conditions affecting the clean-up efficiency. As shown in Figure 3a, with the PEP SPE clean-up (S1, S2, S3, and S4 treatment), 30%, 51%, 52%, and 70% of the analytes were in the recovery of 70–120%, respectively. With the HLB SPE cleanup (S5, S6, S7, and S8 treatment), 57%, 49%, 64%, and 47% of the analytes were in the recovery of 70–120%, respectively. The recoveries of the target compounds that could not meet the recovery requirements under different types of clean-up conditions were displayed in Figure 3b. The results may be due to the fact that the target analytes have broad physical and chemical properties (log Kow: −1.2–6.37), therefore, very hydrophobic analytes were too strongly retained to be eluted with ACN or methanol (MEA) (Figure 3b). In the case of PRiME HLB, the recoveries of 85% of the analytes were in the range of 70–120%, and the recoveries of 14% of the analytes were 60–70%. Thus, we preliminarily adopted PRiME HLB SPE for purification based on the consideration of recoveries. 

In this next experiment, different volumes of eluent were optimized including 1 mL, 2 mL, 3 mL, and 4 mL. The results showed that 85% of the analytes were in the recovery of 70–120% with only 3 mL eluent volumes (Figure 3c). Therefore, 3.0 mL was chosen as the elution volume for PRiME HLB in order to save organic solvents.

Conventional SPE procedures consist of four steps: pre-equilibrating, loading, rinsing, and eluting steps for purification of the sample. The passthrough PRiME HLB column consists of two steps: loading the extraction solution onto the cartridges, and then collecting the eluates. Therefore, this method avoids the tedious steps and reduces solvent consumption and saving in comparison with traditional SPE [22]. 

### 2.3. Validation of the Proposed Method

The validation results of the analytical method in terms of linearity, sensitivity, precision, and accuracy are presented in Table 2 and Table S2. Good linearities (R^2^ > 0.9902) were observed in solvent- and matrix-matched calibration curves. The LOQs of all the analytes were 0.05–5.0 μg/kg. Our results showed that the selectivity and linearity were favorable for the residue analysis at all target concentrations of the pesticides.

All analytes were pre–spiked into samples at the concentration levels of 0.05, 0.5, 5.0, and 50.0 μg/kg (*n* = 5). The average recovery values ranged from 50.4% to 117.4%, with the intra-RSDs being less than 19.8% in grass carp samples. The average recovery values ranged from 60.5% to 118.6%, with intra-RSDs being less than 19.5% in prawn samples. The inter-RSD of the method ranged from 0.5% to 19.1% in grass carp, and 0.3% to 18.5% in prawn, respectively. Our results showed that the recoveries and RSDs can meet the requirements of residue analysis. The matrix effects were assessed by comparing the slope of the matrix standard curve to the solvent standard calibration curve. The ME ranged from 9% to 115% in grass carp, and ranged from 10% to 166% in prawn. The reason for the difference in matrix effect may be related to the physicochemical properties of analytes and the texted matrix, which was observed in other studies [37,38,39]. To eliminate the effect of matrix effects, the matrix-matched calibration curve was selected for the quantitative analysis. Therefore, the developed method can meet the requirements of pesticide residues in grass carp and prawn samples.

### 2.4. Real Samples Analysis and Health Risk Assessment

Of the pesticides studied, three pesticides were detected at values greater than the LOQ. The concentration of dinuron from Tianjin was detected among all samples, with 0.92 μg/kg maximum concentration. Prometryn were detected in all samples from Dalian, and the concentration ranged from 0.05 to 0.06 μg/kg. The concentration of atrazine ranged from LOQ to 0.05 μg/kg in Tianjin, from LOQ to 0.58 μg/kg in Dalian, and from LOQ to 0.28 μg/kg in Qingdao. In the study, dinuron (log Know = 2.87, BCF = 9.7), prometryn (log Know = 3.34, BCF = 85), and atrazine (log Know = 2.7, BCF = 4.3) were detected in samples, probably because of its high log Kow and BCF values. Similarly, dinuron were detected in rice–crayfish systems in Jiangsu Province, with the highest value being 11.5 μg/kg [40]. Prometryn also were detected in the shellfish samples and the concentration ranged from 1.0 μg/kg to 33.6 μg/kg in coastal areas in China [26]. The concentrations of atrazine in aquatic organisms in the Xiangshan Harbor ranged from 2.37 to 39.2 μg/kg (dw), with an average concentration of 13.2 ± 11.8 μg/kg [41]. 

The risk assessment of detected pesticides in prawn samples was shown in Table S3. The acute and chronic risk quotients were calculated, ranging from 0.0029% to 0.0288%, and 0.0002% to 0.0289%, respectively. This indicated that there was not a significant risk to human health.

## 3. Materials and Methods

### 3.1. Reagents and Chemicals 

High purity (>98%) standards of all 90 pesticides and metabolites were purchased from China Standard Material Center (BeiJing, China), Dr. Ehrenstorfer (Augsburg, Germany), Sigma–Aldrich (St. Louis, MO, USA), Fluka (Buchs, Switzerland), AccuStandard (New Haven, CT, USA), and Wako Pure Chemical Industries Inc. (Osaka, Japan). High performance liquid chromatography (HPLC) grade ACN and FA were supplied by Sigma–Aldrich (Steinheim, Germany). Analytical grade ACN, MEA, sodium chloride (NaCl), and anhydrous magnesium sulfate (MgSO_4_) were obtained from Beihua Fine chemicals Co. (BeiJing, China). Ultra-pure water was obtained from a Milli-Q system (Bedford, MA, USA). SPE column, Oasis HLB was purchased from Waters Corporation (Waters Corp., Milford, MA), and Cleanert PEP, PRiME HLB were obtained from Agela Technologies Inc. (Agela, Tianjin, China). Ordinary nitrogen (N_2_) was purchased from Haike Yuanchang Gas (Beijing, China). The Poroshell 120 PFP chromatographic column was purchased from Agilent Technologies (Santa Clara, CA, USA).

### 3.2. Sample Preparation

Grass carp and prawn samples were selected to validate the method. In addition, prawn samples collected from eastern coastal areas of China were used for the real sample analysis. The areas were mainly distributed in Dalian (Liaoning Province), Tianjin, Yantai (Shandong Province), and Qingdao (Shandong Province). A total of 32 prawn samples comprising 8 of each area were used for this work. The prawn meat was homogenized, and stored at −20 °C for the next analysis. 

### 3.3. Extraction and Cleanup 

An amount of 2.0 g of each sample was weighed into a 10 mL centrifuge tube and extracted with 4 mL of 0.2% FA/ACN solution. The samples were vortexed on the multi-functional vortex mixer at 2500 rpm for 10 min. Subsequently, 0.4 g of MgSO_4_ and 0.1 g of NaCl were added, after which the samples were vortexed for 5 min and centrifuged at 8000 rpm for 5 min. An amount of 2 mL of supernatant was prepared for purification.

Method 1: The supernatant (2.0 mL) was evaporated to dryness at 30 °C and was redissolved in 2 mL loading solution for subsequent clean-up. Before sample loading, Oasis HLB and Cleanert PEP cartridges were preequilibrated with 3 mL of methanol and 3 mL of deionized water. Then, the redissolved extracts were loaded into a SPE cartridge. The cartridge was eluted with different solvents and then the eluent was evaporated to dryness by nitrogen at 30 °C. The extract was adjusted to 1 mL with ACN. After filtration with 0.22 μm nylon syringe filters, the final extracts were transferred into an autosampler vial at −20 °C until analysis.

Method 2: The supernatant (2.0 mL) was passed through a PRiME HLB column and there was no activation requirement for PRiME HLB cartridges. Then, the column was eluted by 3.0 mL of ACN. All of the solutions were collected and evaporated to dryness by nitrogen at 30 °C. The extract was adjusted to 1 mL with ACN. After filtration with 0.22 μm nylon syringe filters, the final extracts were transferred into an autosampler vial at −20 °C until analysis.

### 3.4. LC-MS/MS Analysis

The analysis of 90 pesticides and metabolites was performed on an Agilent 1290 UPLC system coupled to a G6470A triple quadrupole mass spectrometer (Agilent Technologies, Santa Clara, CA, USA). Separation was achieved with a Poroshell PFP column (2.1 × 100 mm, 2.7 µm particle size, Agilent) under 30 °C column temperature. The mobile phase was made up of water and ACN. The gradient of mobile phase is shown in Table S4. The injection volume was 5.0 μL, and the flow in the analysis procedure was 0.3 mL/min. 

MS/MS acquisition was carried out by dMRM mode for 90 pesticides and metabolites and the cycle time was 500 ms. The instrument allows for the simultaneous detection of positive and negative ionization modes without affecting method sensitivity, and 90 pesticides and metabolites were analyzed in a single needle injection and the whole analysis time was 17 min (83 analytes in the positive mode and 7 analytes in the negative mode). Other MS detection conditions were set as follows: sheath gas temperature, 350 °C; sheath gas flow, 11 L/min; drying gas temperature, 350 °C; drying gas flow, 10 L/min; nebulizer, 45 psi; and nozzle voltage, 500 V.

### 3.5. Method Validation

The optimized method was verified to evaluate the applicability of the method according to SANTE guidelines [23]. These parameters included linearity, recovery, RSD, LOQ, and ME. The solvent- and matrix-matched calibration curves were set at concentration levels of 0.05, 0.1, 0.5, 1, 2, 5, 10, 20, and 50 μg/L. Good linearity was observed when the correction coefficient (R^2^) of the analyte was greater than 0.99. Four different spiking concentrations on a single day (intra-day) and over three consecutive days (inter-day) were performed to verify the accuracy and precision of the method. The LOQ was the lowest spiked level of the validation that meets the method criteria for recovery and precision. ME was confirmed by comparing the slope of the matrix standard curve to the solvent standard calibration curve.

### 3.6. Risk Assessment

The acute and chronic risk assessment of consuming these prawn samples was investigated. The results were calculated according to the formula [42]:

IESTI = (HR × LP)/bw

%HQa = IESTI/ARfD × 100

where, IESTI is the estimate of short term intake (mg/kg bw), HR indicates the highest concentration (mg/kg), LP means large portion (g/d), and the large portion of prawn is 300 g/d for the general population [43]. bw is the average weight for the general population (60 kg) [42], %HQa is the acute risk quotient, and ARfD is the acute reference dose (mg/kg bw).

EDI = (STMP × F)/bw

%HQ_C_ = EDI/ADI × 100

Here, EDI is the estimated daily intake (mg/kg bw), STMR indicates the median concentration (mg/kg), F means the average consumption of prawn (g/d), and the average consumption of prawn is 100 g/d for the general population [43]. bw is the average weight for the general population (60 kg) [42], %HQc is the chronic risk quotient, and ADI is the acceptable daily intake (mg/kg bw).

## 4. Conclusions

In this study, a strategy based on the purification of the SPE method combined with HPLC-MS/MS for the analysis of 90 pesticides and metabolites in fish and shrimp was proposed. The developed method, with good linearity (>0.9902), specificity, trueness (50.4–118.6%), precision (<19.8%), and sensitivity (0.05–5.0 μg/kg), could meet the requirements for the simultaneous analysis of 90 pesticides and metabolites. The method had the advantages of simple operation and high sensitivity. The results of this study may be helpful for understanding the residue levels in aquatic biota, and the residues in aquatic biota would help us perform risk assessments.

## Figures and Tables

**Table 2:** Linearity, precision, and accuracy of 90 pesticides and metabolites in grass carp and prawn samples.

Analyte	Linearity *	Grass Carp						Prawn					
		Linearity **	Recovery (Intra-RSD, %)				LOQ	Linearity ***	Recovery (Intra-RSD, %)				LOQ
			0.05 μg/kg	0.5 μg/kg	5 μg/kg	50 μg/kg			0.05 μg/kg	0.5 μg/kg	5 μg/kg	50 μg/kg	
Chlorpyrifos	0.9995	0.9994			77.6 (9.3)	85.3 (16.1)	5.0	0.9996			94.1 (5.2)	81.4 (13.8)	5.0
Phoxim	0.9998	0.9989		105.9 (9.9)	77.7 (4.7)	84.9 (16.9)	0.5	0.9998		88.2 (18.5)	85.2 (8.1)	79.0 (14.2)	0.5
Triazophos	0.9986	0.9999		88 (3.6)	83.4 (3.5)	79.9 (10.2)	0.5	0.9960		90.5 (6.8)	89.8 (4.1)	79.6 (15.9)	0.5
Malathion	0.9926	0.9999		89.7 (5.5)	84.2 (3.3)	89.1 (10.6)	0.5	0.9954		60.5 (3.7)	83.6 (7.6)	78.7 (14.2)	0.5
Phorate	0.9995	0.9998		64.2 (5.2)	65.2 (6.1)	84.3 (19.0)	0.5	0.9997		79.2 (14.3)	78.9 (3.7)	81.2 (15.1)	0.5
Phorate-Sulfone	0.9973	0.9919			76.8 (19.2)	85.5 (14.9)	5.0	0.9956		92.3 (10.9)	83.6 (7.6)	81.9 (13.2)	0.5
Phorate-Sulfoxide	0.9986	0.9999	94.0 (1.2)	94.2 (6.1)	93.8 (2.7)	85.9 (17.5)	0.05	0.9980	92.4 (11.4)	95.6 (4.6)	78.9 (3.7)	81.0 (14.7)	0.05
Isocarbophos	0.9923	0.9998	102.3 (11.1)	90.0 (5.3)	66.9 (12.1)	83.0 (16.4)	0.05	0.9976	85.6 (6.3)	87.3 (3.4)	90.2 (3.9)	82.1 (13.0)	0.05
Profenofos	0.9989	0.9997		83.1 (6.1)	70.4 (7.7)	86.2 (17.8)	0.5	0.9997		64.2 (9.9)	89.5 (4.1)	79.9 (15.2)	0.5
Parathion	0.9985	0.997	93.9 (2.7)	87.3 (3.6)	82.8 (3.8)	83.5 (18.7)	0.05	0.9958		92.5 (2.7)	98.7 (3.9)	80.7 (14.1)	0.5
Parathion–methyl	0.9961	0.9913			96.3 (16.5)	97.0 (14.8)	5.0	0.9976			91.8 (4.2)	81.3 (14.0)	5.0
Methamidophos	0.995	0.9996			64.5 (7.5)	86.3 (17.5)	5.0	0.9999			76.9 (4.1)	112.6 (10.8)	5.0
Acephate	0.9945	0.9972			67.7 (6.1)	98.9 (3.4)	5.0	0.9972			98.7 (4.5)	90.7 (1.5)	5.0
Terbufos	0.9998	0.9959			117.4 (12.3)	85.9 (15.3)	5.0	0.9994			85.3 (5.2)	78.2 (13.1)	5.0
Fosthiazate	0.9943	0.9999	114.4 (3.2)	89.6 (2.9)	85.2 (1.5)	87.9 (13.3)	0.05	0.9989	103.2 (10.5)	89.2 (2.4)	88.9 (3.1)	82.5 (18.5)	0.05
Isazofos	0.9986	0.9999		88.1 (3.6)	82.8 (3.2)	86.8 (16.4)	0.5	0.9966		101.4 (3)	90.9 (4.0)	81.8 (13.3)	0.5
Dichlorvos	0.9998	0.9936			61.8 (15.9)	85.4 (16.0)	5.0	0.9996		84.3 (17.8)	78.6 (8.2)	81.6 (14.8)	0.5
Dipterex	0.9992	0.9911			92.9 (8.2)	83.7 (16.2)	5.0	0.9943		78.9 (7.5)	96.7 (7.0)	101.2 (13.9)	0.5
Omethoate	0.9966	0.9982			72.5 (2.1)	92.5 (7.9)	5.0	0.9957			88.3 (7.4)	83.1 (15.4)	5.0
Carbofuran	0.9910	0.9999	115.9 (13.4)	109.8 (4.0)	115.7 (3.9)	87.9 (13.9)	0.05	0.9972	72.5 (8.3)	100.9 (9.3)	117.2 (3.4)	90.3 (8.4)	0.05
3–hydroxycarbofuran	0.9957	0.9997		85.1 (13.0)	79.4 (4.6)	90.2 (13.8)	0.5	0.9975		78.9 (11.5)	87.2 (3.4)	84.5 (12.9)	0.5
Aldicarb	0.9944	0.9991		76.3 (10.7)	77.0 (10.4)	90.8 (6.5)	0.5	0.9920	81.2 (12.6)	82.3 (7.1)	87.6 (4.3)	82.4 (12.7)	0.05
Aldicarb-Sulfone	0.9986	0.9957		89.5 (11.1)	83.8 (3.9)	84.5 (16.5)	0.5	0.9973	77.5 (15.3)	70.2 (12.9)	75.3 (3.4)	85.3 (10.3)	0.05
Aldicarb-Sulfoxide	0.9947	0.9955			74.9 (8.5)	94.8 (9.3)	5.0	0.9992			79.5 (3.1)	83.2 (13.3)	5.0
Methomyl	0.9948	0.9999		91.2 (7.7)	83.6 (5.9)	101.9 (10.7)	0.5	0.9985	71.6 (6.5)	82.3 (1.1)	85.1 (3.6)	86.4 (13.3)	0.05
Isoprocarb	0.9966	0.9999		87.5 (5.1)	76.9 (6.2)	75.4 (16.1)	0.5	0.9986	116.2 (10.7)	74.6 (3.7)	84.6 (4.2)	82.8 (13.0)	0.05
Fenpropathrin	0.9996	0.9946			83.6 (7.3)	97.5 (5.9)	5.0	0.9979			94 (8.1)	84.3 (12.4)	5.0
Chloantraniliprole	0.9991	0.9988		78.5 (12.4)	71.4 (4.8)	90.8 (9.8)	0.5	0.9994		76.7 (5.2)	78.2 (14.8)	92.8 (5.6)	0.5
Flubendiamide	0.9998	0.9997		108.9 (9.4)	81.9 (16.5)	90.5 (11.4)	0.5	0.9998		76.1 (12.3)	92.7 (1.1)	85.4 (11.8)	0.5
Acetamiprid	0.9936	0.9989		57.5 (4.9)	75.9 (5.8)	81.4 (16.4)	0.5	0.9974			83.3 (3.5)	89.8 (13.3)	5.0
Imidacloprid	0.9982	0.9989		79.9 (9.7)	78.3 (2.7)	95.1 (9.0)	0.5	0.9997		89.3 (13.5)	77.3 (7.7)	82.1 (14.7)	0.5
Thiamethoxam	0.9945	0.9979		101.8 (11.7)	79.9 (3.6)	75.2 (13.8)	0.5	0.9994		77.2 (19.5)	84.6 (6.8)	78.5 (16.9)	0.5
Clothianidin	0.9926	0.9945		63.9 (15.8)	82.4 (1.7)	95.1 (5.2)	0.5	0.9986		75.5 (18.9)	85.4 (2.9)	82.6 (15.3)	0.5
Fipronil	0.9994	0.9996		90.6 (2.9)	80.3 (3.6)	90.3 (12.9)	0.5	0.9998		90.2 (2.8)	89.9 (4.6)	79.3 (15.1)	0.5
Fipronil-Sufone	0.9973	0.9999		90.6 (4.6)	70.8 (13.0)	83.2 (17.2)	0.5	0.9984		93.6 (8.6)	90.2 (4.8)	86.7 (14.9)	0.5
Fipronil-Sufoxide	0.9997	0.9999		91.3 (7.4)	70.2 (9.6)	84.8 (15.1)	0.5	0.9998		76.1 (14.9)	83.1 (7.7)	86.6 (12.1)	0.5
Buprofezin	0.9998	0.9999	101.4 (5.2)	84.5 (4.6)	70.1 (7.7)	85.9 (15.8)	0.05	0.9998	95.2 (7.4)	89.2 (0.2)	87.8 (2.6)	84.2 (13.9)	0.05
Hexaflumuron	0.9984	0.9959		109.7 (17.4)	97.5 (10.3)	99.7 (14.8)	0.5	0.9995			94.8 (9.4)	76.9 (14.1)	5.0
Hydroxy Chlorothalonil	0.9875	0.9948			83.7 (9.7)	74.8 (10.6)	5.0	0.9971			70.4 (4.1)	82.1 (13.2)	5.0
Tricyclazole	0.9978	0.9999	103.0 (6.6)	86.5 (4.0)	75.1 (2.6)	96.6 (10.0)	0.05	0.9971	110.2 (4.6)	91.5 (2.1)	85.8 (3.8)	87.8 (9.9)	0.05
Propiconazole	0.9993	0.9999		94.8 (3.9)	78.5 (6.2)	90.6 (15.6)	0.5	0.9991		84.5 (7.2)	89.2 (3.1)	82.9 (12.1)	0.5
Difenoconazole	0.9995	0.9997	97.7 (6.3)	104.2 (7.6)	75.6 (6.1)	88.0 (14.2)	0.05	0.9997	108.8 (11.9)	90.2 (3.8)	86.6 (6.7)	84.3 (12.6)	0.05
Triadimefon	0.9997	0.9993		89.4 (3.4)	87.3 (2.7)	86.7 (13.6)	0.5	0.9980		89.5 (4.0)	80.5 (3.3)	83.2 (13.4)	0.5
Hexaconazole	0.9995	0.9999		90.4 (3.0)	82.3 (5.4)	96.0 (17.3)	0.5	0.9989		85.2 (4.5)	87.3 (2.7)	78.4 (13.1)	0.5
Epoxiconazole	0.9995	0.9999		97.7 (3.5)	76.0 (10.9)	97.2 (14.8)	0.5	0.9992		92.5 (3.8)	87.1 (3.3)	84.6 (10.3)	0.5
Tebuconazole	0.9995	0.9996		90.3 (4.2)	81.6 (5.8)	83.5 (19.8)	0.5	0.9991		91.3 (1.2)	86.5 (3.8)	84.8 (13.7)	0.5
Carbendazim	0.9951	0.9983	92.6 (7.6)	65.3 (15.3)	72.8 (9.1)	84.7 (16.5)	0.05	0.9995	101 (11.1)	90.1 (18.2)	91.6 (3.0)	84.2 (8.3)	0.05
Procymidone	0.9991	0.9975			81.9 (4.6)	97.4 (2.0)	5.0	0.9995		83.3 (10.6)	92.7 (4.3)	84.4 (14.7)	0.5
Azoxystrobin	0.997	0.9999		85.7 (9.6)	91.8 (2.8)	93.1 (11.3)	0.5	0.9995	101.3 (5.1)	86.9 (3.1)	90.2 (3.8)	83.6 (19.0)	0.05
Pyraclostrobine	0.9945	0.9999	89.4 (8.3)	84.4 (3.3)	86.6 (4.8)	84.9 (17.2)	0.05	0.9999		89.2 (7.8)	83.9 (14.3)	82.8 (13.4)	0.5
Pyrimethanil	0.9986	0.9999	90.6 (3.2)	80.6 (3.5)	70.4 (6.7)	99.1 (4.8)	0.05	0.9991	107.5 (17.6)	88.5 (2.9)	88.4 (2.6)	84.6 (15.5)	0.05
Isoprothiolane	0.9982	0.9999	104.9 (9.5)	94.0 (3.9)	79.3 (6.2)	87.5 (14.2)	0.05	0.9986	93.1 (9.9)	88.1 (1.9)	88.6 (3.1)	83.2 (13.0)	0.05
Alachlor	0.9997	0.999		94.6 (17.5)	62.3 (18.3)	87.5 (14.3)	0.5	0.9998		92.2 (15.8)	84.9 (5.5)	85.9 (12.5)	0.5
Acetochlor	0.9971	0.9966		97.5 (8.2)	92.3 (1.6)	107.3 (5.5)	0.5	0.9989		81.2 (5.1)	87.2 (3.5)	82.8 (14.2)	0.5
Bensulfuron methyl	0.9993	0.9998	105.7 (4.2)	89.9 (4.5)	88.5 (18.1)	84.8 (6.2)	0.05	0.9998	94.2 (13.1)	85.4 (3.7)	85.2 (3.1)	82.8 (2.9)	0.05
Pyrazosulfuron-ethyl	0.9981	0.9999		86.8 (3.3)	63.9 (16.7)	89.3 (13.8)	0.5	0.9986		91.6 (4.0)	89.2 (3.4)	78.3 (13.4)	0.5
Tribenuron-methyl	0.993	0.9998		75.2 (4.9)	65.0 (3.5)	85.6 (17.9)	0.5	0.9998		80.2 (11.1)	73.2 (4.1)	83.0 (12.7)	0.5
Atrazine	0.9993	0.9999		89.9 (4.9)	74.9 (6.1)	84.5 (15.0)	0.5	0.9992	95.6 (5.4)	88.6 (1.6)	87.8 (3.1)	80.6 (14.1)	0.05
Desethylatrazine	0.9989	0.9997		91.0 (3.5)	81.5 (2.1)	86.5 (16.2)	0.5	0.9999	96.2 (16.5)	89.5 (3.7)	84.7 (5.4)	83.7 (12.6)	0.05
Desisopropylatrazine	0.998	0.9977		79.7 (14.1)	71.7 (5.2)	98.0 (15.3)	0.5	0.9995		82.2 (10.7)	83.4 (3.8)	81.1 (15.1)	0.5
Hydroxyatrazine	0.998	0.9992		70.5 (6.4)	61.9 (2.8)	95.6 (6.9)	0.5	0.9990	89.5 (16.5)	64.2 (10.1)	68.2 (10.4)	83.1 (13.8)	0.05
Pendimethalin	0.9994	0.9988		109.0 (9.6)	71.8 (5.7)	96.3 (9.6)	0.5	0.9999		81.5 (15.8)	91.5 (4.8)	85.0 (11.6)	0.5
Clomazone	0.9986	0.9999	99.5 (10.9)	88.1 (4.7)	79.2 (3.9)	87.9 (12.1)	0.05	0.9997	107 (12.4)	84.5 (5.0)	85.5 (3.0)	83.9 (2.4)	0.05
Propargite	0.9979	0.9924		79.3 (13.9)	62.9 (12.6)	92.6 (14.8)	0.50	0.9997			76.8 (8.7)	92.0 (11.4)	5.0
Pyridaben	0.9907	0.9989	84.7 (17.1)	68.8 (19.8)	60.3 (13.4)	104.4 (8.8)	0.05	0.9993	115.8 (7.7)	88.2 (8.7)	82.9 (9.0)	88.7 (17.8)	0.05
Paclobutrazol	0.9998	0.9999	92.6 (19.4)	92.9 (5.3)	89.5 (2.2)	83.7 (4.8)	0.05	0.9995	98.8 (11.3)	85.6 (5.2)	88.2 (2.7)	84.5 (17.6)	0.05
Forchlorfenuron	0.9954	0.9999	95.5 (7.1)	79.4 (3.2)	73.0 (3.3)	100.8 (16.9)	0.05	0.9949	76.5 (4.5)	74.2 (1.8)	77.4 (2.8)	84.4 (12.9)	0.05
Carbaryl	0.9951	0.9999		82.1 (4.9)	86.9 (5.8)	88.9 (12.1)	0.5	0.9976		68.5 (4.4)	86.1 (3.1)	88.0 (13.3)	0.5
Indoxacarb	0.9995	0.9998		99.7 (6.0)	60.7 (10.3)	98.3 (17.9)	0.5	0.9995		90.2 (7.7)	95.9 (4.0)	78.0 (12.8)	0.5
Dinotefuran	0.998	0.9987		98.2 (19.4)	74.9 (3.4)	84.6 (17.3)	0.5	0.9999		76.1 (17.8)	70.5 (5.7)	82.5 (13.9)	0.5
Emamectin-benzoate b1a	0.9996	0.9997		99.2 (18.2)	72.8 (10.3)	96.0 (14.0)	0.5	0.9999		79.2 (6.5)	70.2 (5.8)	84.6 (14.6)	0.5
Emamectin-benzoate b1b	0.9983	0.9992		91.6 (10.5)	77.3 (8.7)	90.6 (14.2)	0.5	0.9993			74.6 (6.3)	82.6 (12.2)	5.0
Methoxyfenozide	0.9993	0.9999		94.2 (12.8)	82.1 (3.4)	90.2 (3.8)	0.5	0.9991		92.3 (1.8)	90.7 (3.4)	75.2 (16.8)	0.5
Prothioconazole	0.9995	0.9933			82.8 (9.1)	84 (17.4)	5.0	0.9997			80.9 (6.2)	84.5 (11.5)	5.0
Ametoctradin	0.9996	0.9999		88.4 (3.3)	73.5 (4.8)	103.5 (8.9)	0.5	0.9997		85.6 (3.5)	87.8 (2.9)	113.4 (13.6)	0.5
Boscalid	0.9901	0.9947			72.7 (17.3)	101.4 (8.8)	5.0	0.9933		83.5 (7.4)	85.9 (3.1)	83.6 (12.3)	0.5
Flutolanil	0.9975	0.9999		92.8 (4.2)	85.7 (3.6)	90.5 (10.1)	0.5	0.9996		82.6 (5.5)	86.9 (2.0)	86.3 (10.4)	0.5
Fomesafen	0.9992	0.9991		105.4 (13.2)	62.2 (19.1)	99.8 (15.5)	0.5	0.9995			92.1 (4.4)	83.3 (12.8)	5.0
Prometryn	0.9997	0.9999	103.6 (6.4)	86.4 (3.9)	72.6 (8.5)	91.9 (7.6)	0.05	0.9998	93.4 (7.5)	87.2 (1.1)	87.7 (3.2)	84.9 (16.3)	0.05
Imazethapyr	0.9997	0.9998		76.8 (4.3)	66.8 (3.3)	97.1 (5.5)	0.5	0.9981		60.9 (8.0)	61.8 (5.4)	82.2 (13.9)	0.5
Nicosulfuron	0.9994	0.9998	105.7 (4.2)	89.8 (3.6)	87.7 (6.5)	85.3 (14.6)	0.05	0.9998	94.2 (13.1)	85.2 (3.7)	86.0 (2.6)	83.1 (13.6)	0.05
Sulfometuron-methyl	0.9989	0.9999		87.1 (3.4)	82.6 (1.8)	89.3 (13.8)	0.5	0.9994	112 (14.9)	82.1 (1.4)	84.9 (3.6)	97.4 (13.4)	0.05
Mesotrione	0.9919	0.995		60.3 (16.9)	75.6 (6.8)	100 (13.3)	0.5	0.9839		62.5 (5.6)	63.3 (8.2)	82.2 (13.6)	0.5
Diuron	0.9948	0.9999		80.7 (5.6)	73.3 (10.5)	76.5 (15.6)	0.5	0.9939	108.5 (7.2)	87.6 (1.1)	88.7 (2.9)	81.1 (12.8)	0.05
Quinclorac	0.9996	0.9993		50.4 (15.3)	52.5 (2.6)	88.9 (12.3)	0.5	0.9974		75.7 (11.7)	77.2 (5.9)	87.9 (10.5)	0.5
Pretilachlor	0.9988	0.9982	100.2 (8.1)	84.8 (3.7)	87.6 (3.5)	98.4 (13.2)	0.05	0.9990	105.4 (10.8)	88.2 (3.4)	87.9 (2.1)	75.5 (12.8)	0.05
Metolachlor	0.9983	0.9998	105.0 (4.6)	87.5 (2.2)	71.1 (11.8)	85.3 (17.5)	0.05	0.9996	118.6 (9.1)	90.1 (3.8)	88.8 (2.8)	84.3 (12.7)	0.05
Flumetsulam	0.9938	0.9999	100.3 (9.2)	89.3 (4.5)	77.5 (4.9)	86.4 (16.5)	0.05	0.9864	97.7 (6.8)	82.4 (4.7)	83.2 (4.4)	85.9 (8.3)	0.05
Penoxsulam	0.9972	0.9999	104.5 (6.3)	93.3 (1.7)	83.8 (3.1)	88.7 (13.1)	0.05	0.9990	109.9 (7.6)	91.8 (2.3)	89.6 (4.1)	86.0 (10.5)	0.05
Saflufenacil	0.9991	0.9992		86.9 (4.2)	70.6 (14.6)	100.3 (16.7)	0.5	0.9995		85.2 (1.6)	91.9 (4.9)	98.9 (12.5)	0.5

* represents solvent calibration curves; ** represents matrix-matched calibration curves in grass carp; *** represents matrix-matched calibration curves in prawn.

## Data Availability

Not applicable.

## Supplementary Materials

The following supporting information can be downloaded at: https://www.mdpi.com/article/10.3390/molecules28104235/s1, Figure S1: Total ion response through MRM mode and dynamic MRM mode (red: MRM; blue, dMRM mode); Table S1: Experimental parameters and HPLC–MS/MS conditions of 90 pesticides and metabolites in ESI; Table S2: Inter RSDs and matrix effect of 90 pesticides and metabolites in grass carp and prawn samples; Table S3: Acute and chronic risk assessment of detected pesticides in prawn samples; Table S4: The gradient of mobile phase for the instrumental analysis by HPLC-MS/MS.

## Sample Availability

Samples of the compounds are available from the authors.
