# Supplementary material for: Highly-Selective Analytical Strategy for 90 Pesticides and Metabolites Residues in Fish and Shrimp Samples
Source: Molecules. 2023 May 22;28(10):4235. doi: 10.3390/molecules28104235 (PMC10222193; doi:10.3390/molecules28104235)
Supplement: Supplementary file 1 [file molecules-28-04235-s001.zip › molecules-2394212-supplementary.pdf]

Highly-selective analytical strategy for 90 pesticides and metabolites residues in fish and shrimp samples

Yage Guo[1], Jun Xie[1], Fengshou Dong[1], Xiaohu Wu[1], Xinglu Pan[1], Yongquan Zheng[1], Jie Zhang[2,*], Jun Xu[1,*]

1 State Key Laboratory for Biology of Plant Diseases and Insect Pests, Institute of Plant Protection, Chinese Academy of Agricultural Sciences, Beijing, 100193, P. R. China

2 Institute of Zoology, Chinese Academy of Sciences, Beijing 100101, P. R. China

*Correspondence:

Jun Xu,

E-mail address: junxu1@ippcaas.cn

Tel.: +86 10 62815938; fax: +86 10 62815938

Jie Zhang,

E-mail address: zhangjie@ioz.ac.cn

List of Figures and Tables

Table S1 Experimental parameters and HPLC–MS/MS conditions of 90 pesticides and metabolites in ESI

Table S2 Inter RSDs and matrix effect of 90 pesticides and metabolites in grass carp and prawn samples

Table S3 Acute and chronic risk assessment of detected pesticides in prawn samples

Table S4 The gradient of mobile phase for the instrumental analysis by HPLC-MS/MS.

Figure S1 Total ion response through MRM mode and dynamic MRM mode (red: MRM; blue, dMRM mode)

Table S1 Experimental parameters and HPLC–MS/MS conditions of 90 pesticides and metabolites in ESI

Analytes	mode	Retention Time/min	CAS	Precursor ion(m/z)	Frag (V)	Quantitative ion（m/z）	CE (V) Quant	Qualitative ion（m/z）	CE (V) Qual
Chlorpyrifos	+	13.22	2921-90-2	349.9	90	197.9	22	97	36
Phoxim	+	12.12	14816-18-3	299.1	80	129.2	10	77	35
Triazophos	+	9.57	24017-47-8	314.1	110	162	21	119.1	41
Malathion	+	9.49	121-75-5	331	80	126.9	6	125.1	34
Phorate	+	11.35	298-02-2	261.1	53	199	3	75.1	9
PhorateSulfone	+	6.88	2588/4/7	293	61	171	8	97	42
Phorat-sulfoxide	+	4.38	2588/3/6	277	58	199	7	97	42
Isocarbophos	+	6.85	24353-61-5	230.9	105	121.1	21	65.1	47
Profenofos	+	11.47	41198-08-7	373.1	110	344.8	11	128.1	55
Parathion	+	9.58	56-38-2	314	75	162.1	21	119.2	43
Parathion-methyl	+	9.90	298-00-0	281	85	160.1	15	187.9	5
Methamidophos	+	12.92	10256-92-6	142	48	125	13	94	13
Acephate	+	1.09	30560-19-1	143	100	95	17	65	45
Terbufos	+	12.92	13071-79-9	233	70	198.9	2	186.9	5
Isofenphos-methyl	+	11.19	99675-03-3	354.1	90	252.8	16	213.9	33
Fosthiazate	+	4.29	98886-88-3	284	90	228.1	5	104.1	20
Isazofos	+	9.83	42509-80-8	314	79	162	15	120	30
Dichlorvos	+	3.70	62-73-7	221	100	109	12	79	24
Dipterex	+	2.64	52-68-6	257	105	221	7	109	15
Omethoate	+	1.09	1113-02-6	213.9	70	125	22	109	32
Carbofuran	+	3.96	99485-76-4	222	66	164.8	13	123	21
3-hydroxycarbofuran	+	2.71	16655-82-6	238.1	109	163.1	9	107.1	32
Aldicarb	+	3.29	116-06-3	116	85	89.1	7	70.1	7
Aldicarb-Sulfone	+	2.47	1646-88-4	223.1	110	86.1	13	76.1	5
Aldicarb-Sulfoxide	+	2.34	1646-87-3	207	50	132.1	1	89.1	11
Methomyl	+	2.54	16752-77-5	163.1	60	106.1	5	88.1	5
Isoprocarb	+	4.91	2631-40-5	194.1	86	95	13	137.1	5
Fenpropathrin	+	13.21	39515-41-8	350.2	66	125.1	15	97.1	37
Chloantraniliprole	+	6.17	500008-45-7	482	104	284	11	112	81
Flubendiamide	—	11.69	272451-65-7	681.01	120	271.9	13	254	29
Acetamiprid	+	3.04	135410-20-7	223.1	120	126	21	56.2	17
Imidacloprid	+	2.90	138261-41-3	256.1	120	209	13	175.1	21
Thiamethoxam	+	2.62	153719-23-4	292	52	211	5	181	25
Clothianidin	+	2.81	210880-92-5	250	77	131.8	20	168.7	10
Fipronil	—	12.16	120068-37-3	435	116	329.9	13	249.9	29
Fipronil-Sufone	—	13.43	120068-36-2	450.9	116	414.8	15	282	29
Fipronil-Sufoxide	—	13.27	120067-83-6	418.9	98	261.8	10	418.9	30
Buprofezin	+	4.48	69327-76-0	306.2	95	201.1	9	57.1	25
Hexaflumuron	—	13.06	86479-06-3	458.9	115	438.9	9	175	41
Hydroxy Chlorothalonil	—	14.00	28343-61-5	244.9	75	181.9	33	174.9	29

Tricyclazole	+	3.10	41814-78-2	190.1	115	163.1	25	136	33
Propiconazole	+	9.38	60207-90-1	342.08	91	158.9	37	69.1	21
Difenoconazole	+	10.96	119446-68-3	406	136	250.9	28	188	52
Triadimefon	+	8.54	43121-43-3	294.1	105	69.1	21	197.1	15
Hexaconazole	+	8.55	79983-71-4	314.08	120	70.1	17	158.9	41
Epoxiconazole	+	8.67	135319-73-2	330	115	121.1	23	101	59
Tebuconazole	+	8.89	107534-96-3	308.1	120	70.1	25	125.1	45
Carbendazim	+	1.10	120068-37-3	192.1	100	160	21	132.1	37
Procymidone	+	9.52	32809-16-8	284	88	256.1	17	95.1	25
Azoxystrobin	+	8.53	131860-33-8	404.1	105	372.1	13	329.1	33
Pyraclostrobine	+	11.60	175013-18-0	388.1	68	194.1	9	163.1	25
Pyrimethanil	+	3.19	53112-28-0	200.1	130	107.1	27	82.1	30
Isoprothiolane	+	8.85	50512-35-1	291.1	76	231	9	189	21
Alachlor	+	12.50	15972-60-8	270.1	71	238.1	9	162	23
Acetochlor	+	8.62	34256-82-1	270.1	75	224	7	148	19
Bensulfuron methyl	+	6.61	83055-99-6	411.1	90	182	20	149.1	20
Pyrazosulfuron-ethyl	+	8.21	93697-74-6	415.1	81	182	20	83	66
Tribenuron-methyl	+	7.02	101200-48-0	396.1	75	181.1	19	155.1	13
Atrazine	+	4.43	1912-24-9	216.1	107	174	19	96.1	27
Desethylatrazine	+	2.79	6190-65-4	188	98	146.1	17	104	30
Desisopropylatrazine	+	2.50	1007-28-9	174	96	96.1	20	104	26
Hydroxyatrazine	+	1.10	2163-68-0	198.1	107	156	19	86.1	26
Pendimethalin	+	13.40	40478-42-1	282.1	76	212	8	194	18
Clomazone	+	5.71	81777-89-1	240	91	125	21	89.1	57
Propargite	+	13.50	2312-35-8	368.1	62	231.1	7	175.1	14
Pyridaben	+	13.59	96489-71-3	365.1	73	309	11	147.1	27
Paclobutrazol	+	6.72	76738-62-0	294.1	76	70.1	21	125	43
Forchlorfenuron	+	5.58	68157-60-8	248	82	129	17	93.1	44
Carbaryl	+	4.68	63-25-2	202	65	145	2	127.1	28
Indoxacarb	+	13.03	144171-61-9	528.1	105	249	16	203	47
Dinotefuran	+	2.36	165252-70-0	203.12	60	129.1	10	87.1	19
Emamectin-benzoate b1a	+	8.37	155569-91-8	886.4	140	157.9	50	81.6	80
Emamectin-benzoate b1b	+	7.48	155569-91-8	872.6	140	158	50	82	80
Methoxyfenozide	+	9.53	161050-58-4	313.1	58	149.1	11	91.1	46
Prothioconazole	+	9.38	178928-70-6	344	95	154	33	189.1	23
Ametoctradin	+	7.24	865318-97-4	276.2	110	176	46	149	45
Boscalid	+	8.71	188425-85-6	343	85	307.1	21	272	37
Flutolanil	+	10.15	66332-96-5	324.1	99	242.1	19	262.1	29
Fomesafen	—	11.26	72178-02-0	437	119	195	43	222	35
Prometryn	+	3.03	7287-19-6	242.2	125	158.1	25	200.1	19
Imazethapyr	+	3.19	81335-77-5	290.2	119	245.1	22	177	30
Nicosulfuron	+	6.61	686-897-5	411.1	106	182	20	213	14
Sulfometuron-methyl	+	4.60	74222-97-2	365.1	78	150.1	17	107.1	51
Mesotrione	+	2.70	104206-82-8	293	133	214	37	202	36

Diuron	+	5.56	330-54-1	233	74	72.1	20	159.9	32
Pretilachlor	+	11.83	51218-49-6	312.1	75	252.1	17	176.1	32
Metolachlor	+	8.25	51218-45-2	284.1	101	252.1	15	176.1	29
Flumetsulam	+	3.28	98967-40-9	326	104	129	27	109	67
penoxsulam	+	7.53	219714-96-2	484	154	195	31	164	37
Saflufenacil	+	9.32	372137-35-4	501	163	198	55	349	30

Table S2 Inter RSDs and matrix effect of 90 pesticides and metabolites in grass carp and prawn samples

Analytes	grass carp (Inter RSD, %)				prawn (Inter RSD, %)				Matrix effect (%)	
	0.05	0.5	5	50	0.05	0.5	5.0	50	Grass carp	prawn
Chlorpyrifos			10.1	6			12.4	3.1	14	25
Phoxim		9.2	6.7	11.1		0.8	9.0	1.8	17	35
Triazophos		12.0	5.1	11.1		1.3	10.1	10.8	72	76
Malathion		14.2	9.1	9.3		7.2	11.1	9.1	39	48
Phorate		5.3	8.1	7.3		6.5	10.5	6.5	17	25
PhorateSulfone		9.1	3.7	9.1		1.7	9.3	8.9	51	65
Phorat-sulfoxide	10.1	13.4	6.8	11.8	16.4	2.4	10.8	1.1	92	89
Isocarbophos	11.1	11.4	1.3	7.7	16.7	2.3	8.7	7.1	52	66
Profenofos		1.1	5.3	7.0		0.5	9.2	7.0	34	53
Parathion	10.7	10.6	5.1	8.8		0.7	10.4	7.7	85	69
Parathion-methyl			7.5	6.9			8.8	5.7	40	115
Methamidophos			10.8	11.1			14.2	12.2	28	26
Acephate			12.4	8.3			8.2	11.5	9	22
Terbufos			9.0	8.9			14.2	8.1	31	40
Isofenphos-methyl	11.0	15.3	9.4	14.2	5.8	1.2	1.1	14.0	10	12
Fosthiazate		11.7	5.7	11.2		1.4	1.2	1.9	62	97
Isazofos		6.8	11.8	13.2		10.6	12.6	2.0	86	68
Dichlorvos		17.1	13.8	11.5		14.1	18.4	7.7	27	24
Dipterex			16.7	15.0			4.8	9.2	61	63
Omethoate		7.8	4.9	8.1	12.9	2.4	1.2	1.4	20	19
Carbofuran	13.2	6.5	2.2	4.0		2.5	3.9	1.7	54	59
3-hydroxycarbofuran		6.9	7.1	9.7	13.2	3.5	6.0	1.8	15	104
Aldicarb		8.2	3.1	2.0	18.3	2.6	4.1	1.5	47	51
Aldicarb-Sulfone			7.1	3.1			13.9	1.0	11	26
Aldicarb-Sulfoxide		12.9	4.4	12.7	16.1	0.7	2.8	3.1	10	23
Methomyl		11.2	5.1	10.1	12.9	3.0	1.7	8.1	33	86
Isoprocarb			10.5	8.9			6.9	8.4	39	55
Fenpropathrin		15.5	2.9	13.1		2	0.5	2.1	27	16
Chloantraniliprole		10.7	12.6	6.3		2.4	4.3	1.7	80	154
Flubendiamide		9.2	3.9	4.9			1.8	1.3	52	41
Acetamiprid		3.9	9.8	2.2		15.3	9.4	2.1	39	90
Imidacloprid		8.5	6.2	2.3		10.0	11.5	2.3	11	17
Thiamethoxam		8.1	11.9	2.8		8.7	1.6	3.9	15	15
Clothianidin		13.9	14	16.9		6.3	1.9	6.5	22	39
Fipronil		14.6	7.8	12.9		6.2	4.4	4.9	104	88
Fipronil-Sufone		5.2	9.1	9.5		3.0	3.7	8.5	66	54
Fipronil-Sufoxide	18.9	17.0	11.2	16.1	3.8	0.7	1.6	2.2	90	119
Buprofezin		12.3	5.8	9.4			3.6	2.6	45	51
Hexaflumuron		8.0	11.6	11.3			4.7	14.5	47	34
Hydroxy Chlorothalonil	17.2	10.5	7.5	11.9	5.3	2.1	10.6	1.0	11	11

Tricyclazole		12.6	12.1	16.0		4.7	1.6	7.8	60	58
Propiconazole	11.7	10.6	14.6	12.6	5.3	1.4	2.1	1.8	43	39
Difenoconazole		12.7	11.9	13.6		2.1	5.2	15.6	26	30
Triadimefon		12.9	14.1	14.7		2.2	15.3	15.1	30	31
Hexaconazole		15.2	12.3	14.4		1.4	11.3	14.4	30	32
Epoxiconazole		11.4	13.5	14.7		1.5	14.5	5.2	16	34
Tebuconazole	14.7	7.1	7.6	8.2	4.3	6.8	8.2	7.9	31	30
Carbendazim			5.3	13.9		9.0	7.3	4.1	9	13
Procymidone		9.5	6.0	10.9	3.7	1.7	1.1	10.9	73	21
Azoxystrobin	7.4	6.7	6.2	5.1		2.6	6.6	4.8	62	105
Pyraclostrobine	10.2	7.7	12.7	12.3	12.3	3.7	12.5	6.3	31	47
Pyrimethanil	11.2	9.5	5.3	10.3	5.9	10.6	6.0	10.2	39	48
Isoprothiolane		7.8	8.0	11.3		6.1	2.2	11.1	55	63
Alachlor		19.1	8.2	10.3		6.3	6.3	14.5	11	33
Acetochlor		16.9	11.4	17.5	4.5	17.5	12.8	1.4	44	65
Bensulfuron methyl	5.6	6.8	11.0	8.3		1.5	11.9	1.7	115	93
Pyrazosulfuron-ethyl		16.9	4.7	7.0		1.5	1.7	6.6	92	132
Tribenuron-methyl		14.3	9.6	13.0	7.9	3.5	0.9	5.0	29	26
Atrazine		11.4	13.9	14.7	15.2	4.9	2.8	0.9	42	43
Desethylatrazine		16.4	15.8	7.8		16.1	11.9	1.4	27	31
Desisopropylatrazine		10.6	13.9	7.1	19.7	1.4	2.9	12.1	17	38
Hydroxyatrazine		7.2	10.3	9.2		8.0	12.1	4.1	16	20
Pendimethalin	5.7	10.0	6.9	11.7	4.4	1.8	8.3	11.4	11	10
Clomazone		10.6	6.9	10.4			12.1	4.6	86	72
Propargite	9.0	5.9	7.3	12.8	17.2	6.9	15.9	6.4	9	10
Pyridaben	7.4	17.0	13.6	13.7	15.9	4.4	14.7	13.8	12	14
Paclobutrazol	18.6	2.5	6.6	2.0	6.0	1.1	7.8	1.4	44	37
Forchlorfenuron		5.1	1.3	6.5		0.8	1.2	6.1	26	56
Carbaryl		19.0	9.7	7.6		5.2	11.0	6.3	38	43
Indoxacarb		15.1	1.6	8.4		18.5	14.5	9.4	44	72
Dinotefuran		18.6	14.8	15.0		3.4	1.7	8.2	10	17
Emamectin-benzoate bla		11.5	9.9	8.6			8.2	8.9	81	58
Emamectin-benzoate bab		12.9	8.9	13.4		0.3	10.8	3.1	78	64
Methoxyfenozide			3.3	15.8			12.3	4.5	76	70
Prothioconazole		16.2	13.8	11.6		3.2	2.1	1.9	39	35
Ametoctradin			17.4	1.1		6.6	17.3	2.1	73	57
Boscalid		4.8	2.7	8.2		1.5	2.6	8.2	47	59
Flutolanil		4.6	2.2	7.2			7.8	2.9	46	52
Fomesafen	17.6	13.9	12.5	15.2	10.8	17.4	2.8	1.2	93	31
Prometryn		17.4	8.3	14.9		2.8	9.5	9.8	54	54
Imazethapyr	3.2	16.9	11.4	17.5	4.5	14.9	1.0	7.9	58	45
Nicosulfuron		12.6	8.3	16.8	9.1	14.4	1.5	16.9	111	95
Sulfometuron-methyl		8.7	3.9	12.0		11.2	8.3	2.9	70	64
Mesotrione		6.9	0.5	8.5	3.6	1.7	1.0	8.5	9	18

Diuron		5.3	14.7	9.5		1.5	2.0	9.2	85	91
Pretilachlor	16.9	9.5	2.1	9.0	19.8	5.4	2.5	14.5	39	56
Metolachlor	17.3	9.8	7.3	11.5	4.9	2.5	8.1	10.9	48	71
Flumetsulam	8.5	3.1	2.0	4.1	18.3	5.4	3.1	3.9	76	166
Penoxsulam	6.8	13.4	9.7	12.0	5.5	10.1	10.8	8.2	108	133
Saflufenacil		14.5	8.9	12.9		8.1	10.6	5.1	58	166

Table S3 Acute and chronic risk assessment of detected pesticides in prawn samples

Pesticide	Acute risk assessment			Chronic risk assessment		
	ARfD (mg/kg bw)	IESTI (mg/kg bw)	%HQ_a (%)	ADI (mg/kg bw)	EDI (mg/kg bw)	%HQ_C (%)
atrazine	0.1	0.0000029	0.0029	0.02	0.000000087	0.0004
prometryn	-	0.0000003	-	0.04	0.000000087	0.0002
dinuron	0.016	0.0000046	0.0288	0.003	0.000000867	0.0289

ADI and ARfD were obtained from the Joint FAO/WHO Meeting on Pesticide Residues (JMPR) (http://www.fao.org/agriculture/crops/core-themes/theme/pests/lpe/en/).

Table S4 The gradient of mobile phase for the instrumental analysis by HPLC-MS/MS.

	Time (min)	Initial	0.5	1.0	5.0	12.0	12.5	15.0	15.1	17.0
Mobile phase A	water (%)	98%	98%	65%	65%	40%	2%	2%	98%	98%
Mobile phase B	Acetonitrile (%)	2.0%	2.0%	35%	35%	60%	98%	98%	2%	2%

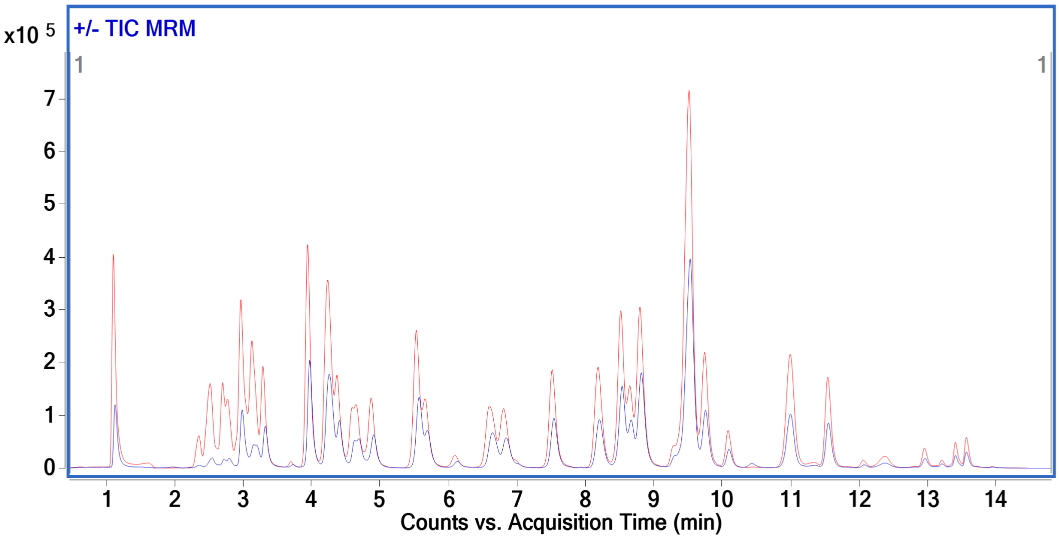

Figure S1 Total ion response through MRM mode and dynamic MRM mode (red: dMRM; blue, MRM mode)
